# Supplementary material for: All-optical control of spin in a 2D van der Waals magnet
Source: Nat Commun. 2022 Oct 10;13:5976. doi: 10.1038/s41467-022-33343-4 (PMC9551086; doi:10.1038/s41467-022-33343-4)
Supplement: Supplementary file 1 — Supplementary Information [file 41467_2022_33343_MOESM1_ESM.pdf]

# Supplementary Information for

## All-optical control of spin in a 2D van der Waals magnet

Maciej Dąbrowski[1*], Shi Guo[1], Mara Strungaru[2], Paul S. Keatley[1], Freddie Withers[1], Elton J. G. Santos[2,3†], Robert J. Hicken[1‡]

[1] *Department of Physics and Astronomy, University of Exeter, EX4 4QL, United Kingdom*

[2] *Institute for Condensed Matter Physics and Complex Systems, School of Physics and Astronomy, The University of Edinburgh, EH9 3FD, United Kingdom*

[3] *Higgs Centre for Theoretical Physics, The University of Edinburgh, EH9 3FD, United Kingdom*

Corresponding authors: *m.k.dabrowski@exeter.ac.uk, †esantos@ed.ac.uk, ‡r.j.hicken@exeter.ac.uk

**This Supplementary Information file includes:**

1. Optical images and photoluminescence of $CrI_3/WSe_2$ heterostructures (Figures S1 - S2).

2. Atomic-force microscopy (AFM) images of $CrI_3$ and $CrI_3/WSe_2$ (Figures S3 - S5).

3. Magnetization reversal, domain structure and optically induced domain formation for isolated bulk $CrI_3$ flakes (Figures S6 - S8).

4. Domain structure and optically induced domain formation and additional AOS for heterostructure $CrI_3(10\,nm)/WSe_2(1\,L)$ (sample 2) (Figures S9 - S13).

5. Magnetization reversal and Kerr images showing the AOS for a $CrI_3(10\,nm)/WSe_2(1\,L)$ heterostructure (sample 3) (Figures S14 - S17).

6. Ultrafast atomistic spin dynamics (Figures S18 - S20, Table 1).

7. Supplementary References.

## 1 Optical images and photoluminescence of $CrI_3/WSe_2$ heterostructures

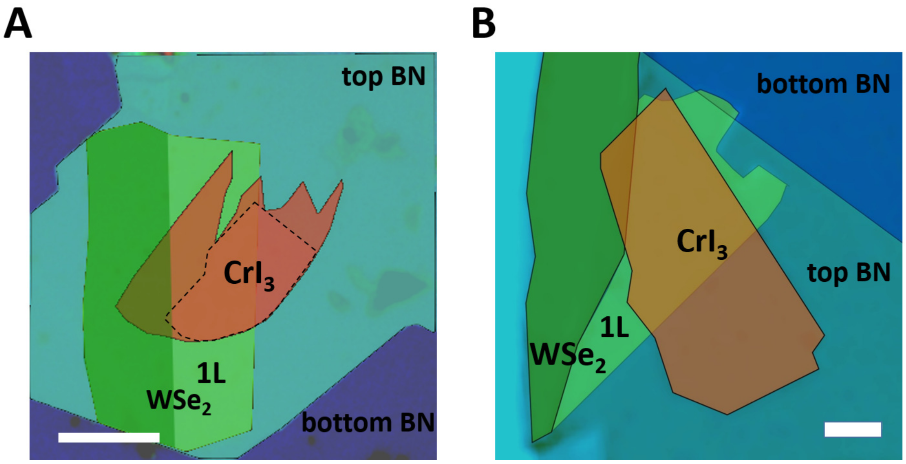


Figure S1: **Optical images of $CrI_3/WSe_2$ heterostructures (samples 2 and 3).** Optical images of sample 2 **(A)** and sample 3 (**B**). The bright green areas correspond to a monolayer (1 L) of $WSe_2$, while darker green corresponds to thicker $WSe_2$. The $CrI_3$ flakes are 10 nm thick in both samples. The dashed black line in **(A)** outlines the area which is uniformly magnetized and can be reversed with the available magnetic field. The scale bars have 5 $\mu$m length.

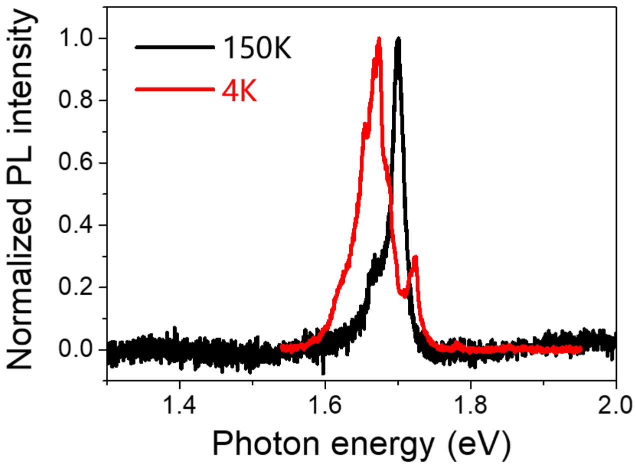


Figure S2: **Photoluminescence of $WSe_2$.** Normalized photoluminescence spectra of a $CrI_3/WSe_2$ heterostructure (sample 2) acquired at 4 K (red line) and 150 K (black line) upon excitation with 405 nm linearly polarized light with average power of 200 $\mu$W. The main peak of the spectra is attributed to the trion peak [1].

## 2 Atomic-force microscopy (AFM) images of $CrI_3$ and $CrI_3/WSe_2$

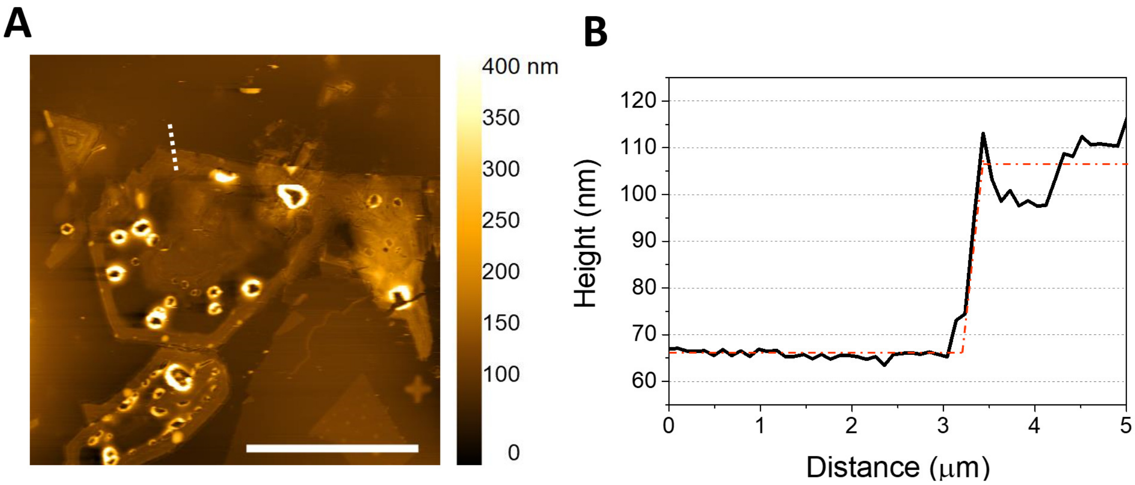


Figure S3: **AFM image of an isolated $CrI_3$ bulk flake of $\sim$40nm thickness exfoliated on $SiO_2$/Si (sample 1).** The scale bar has 20 $\mu$m length.

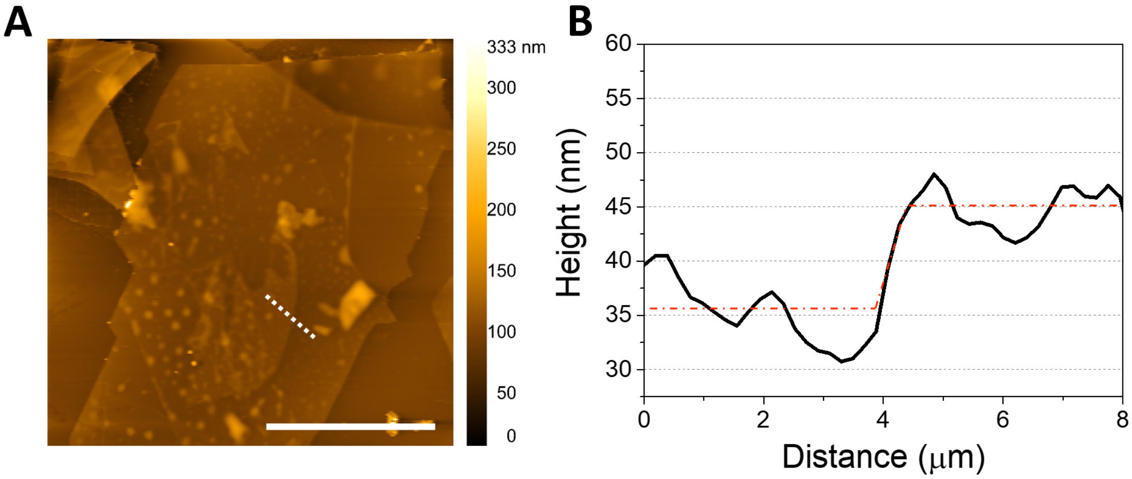


Figure S4: **AFM image of $CrI_3/WSe_2$ encapsulated in h-BN and exfoliated on $SiO_2$/Si (sample 2).** The line profile taken across the edge of the $CrI_3$ flake shows the thickness to be $\sim$ 10nm. The scale bar has 20 $\mu$m length.

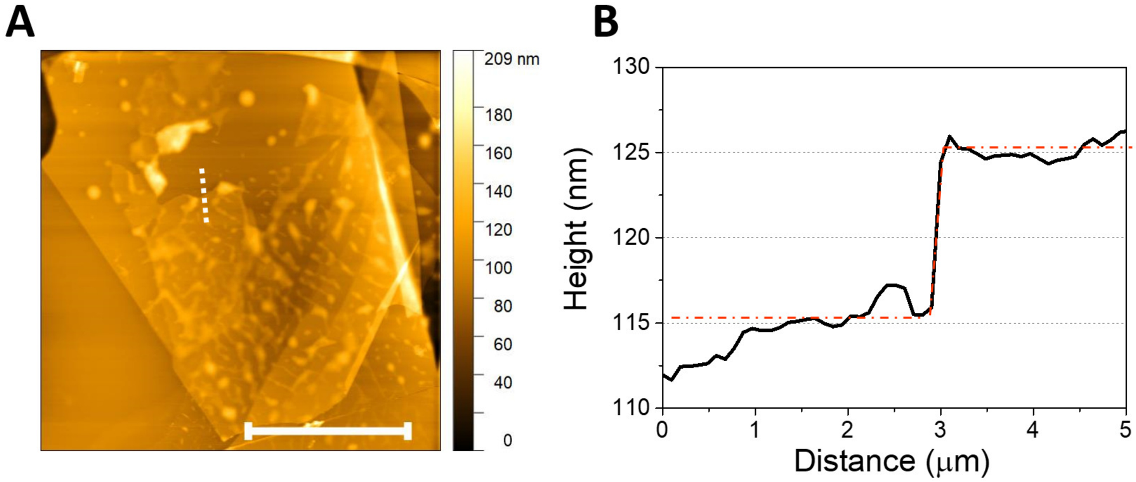


Figure S5: **AFM image of $CrI_3/WSe_2$ encapsulated in h-BN and exfoliated on $SiO_2$/Si (sample 3).** The line profile taken across the edge of the $CrI_3$ flake shows the thickness to be $\sim$ 10nm. The scale bar has 20 $\mu$m length.

## 3 Magnetization reversal, domain structure and optically induced domain formation for isolated bulk $CrI_3$ flakes.

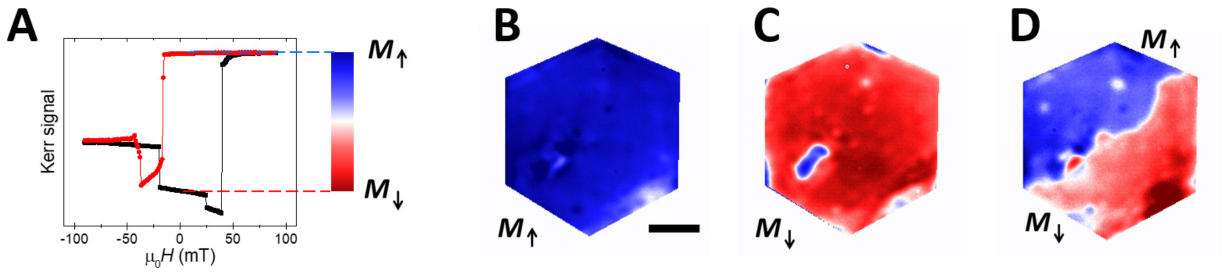


Figure S6: **Magnetization reversal and domain structure of an isolated $\sim$40 nm bulk $CrI_3$ flake (sample 1). (A)** Polar MOKE hysteresis loop as a function of applied magnetic field with the Kerr signal integrated over the entire area of the flake. Kerr images of remanent initial states **(B)** $M_\uparrow$ and **(C)** $M_\downarrow$ obtained after saturation at +100 mT and $-100$ mT, respectively. The magnetization reversal is sensitive to the field sweep rate. Rapid change of the magnetic field from saturation to remanence leads to creation of a domain structure at remanence, as shown in **(D)**, where coexistence of oppositely oriented $M_\uparrow$ and $M_\downarrow$ domains is observed. The measurements were performed at $T = 20$ K. The scale bar has 5 $\mu$m length.

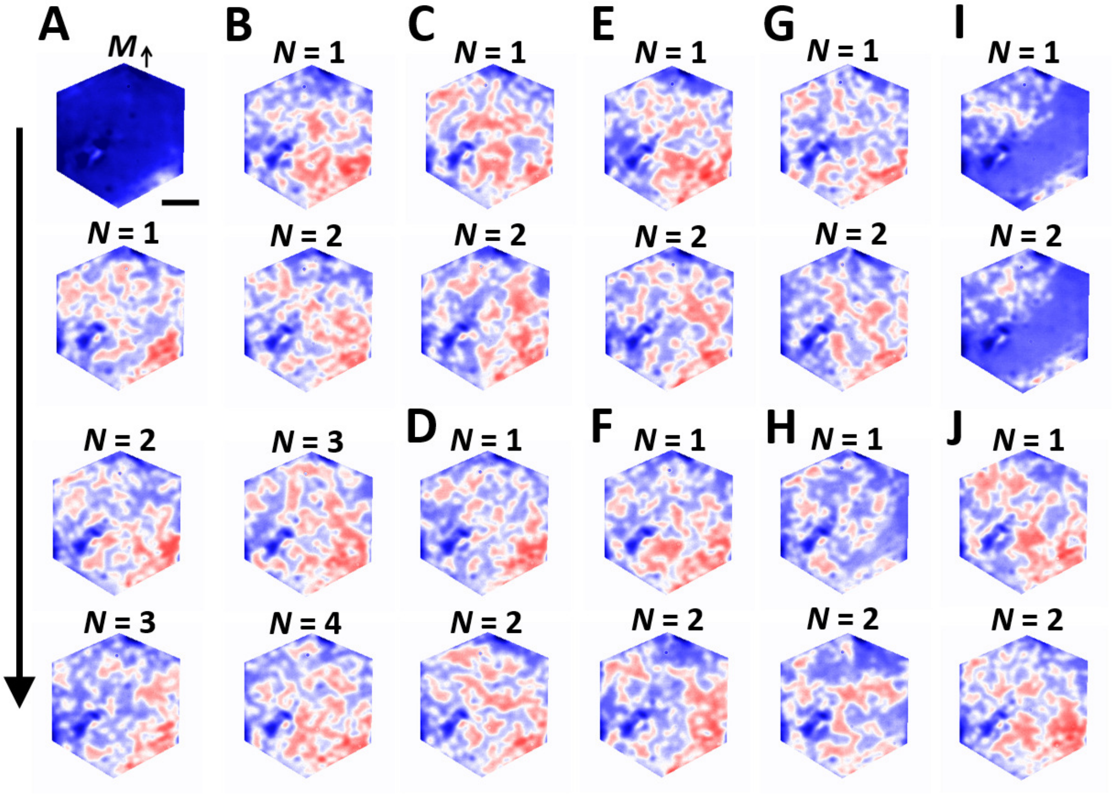


Figure S7: **Demagnetization and optically-induced domain formation for a $CrI_3$ bulk flake (sample 1).** **(A)** The remanent $M_\uparrow$ mono-domain state and changes to its domain structure after exposure to $N$ consecutive single pulses with linear polarization $\pi$ and fluence $F = 3.4\,\mathrm{mJ/cm^2}$. **(B)** similar to **(A)** but starting from the $M_\downarrow$ initial state. **(C)** and **(D)** for $\sigma^+$ circular polarization starting from $M_\uparrow$ and $M_\downarrow$, respectively. **(E)** and **(F)** for $\sigma^-$ circular polarization starting from $M_\uparrow$ and $M_\downarrow$, respectively. Excitation of the $M_\uparrow$ initial state with single $N$ pulses of $\pi$ polarization and fluence of **(G)** $F = 1.8\,\mathrm{mJ/cm^2}$, **(H)** $F = 0.5\,\mathrm{mJ/cm^2}$ and **(I)** $F = 0.25\,\mathrm{mJ/cm^2}$. **(J)** Excitation of the $M_\uparrow$ initial state with $N \times 10^6$ pulses and $F = 2.5\,\mathrm{mJ/cm^2}$. All measurements were performed at $T = 20\,\mathrm{K}$, with photon energy $E = 1.88\,\mathrm{eV}$ , and $\sim 30\,\mathrm{fs}$ pulse duration. The scale bar has $5\,\mu\mathrm{m}$ length.

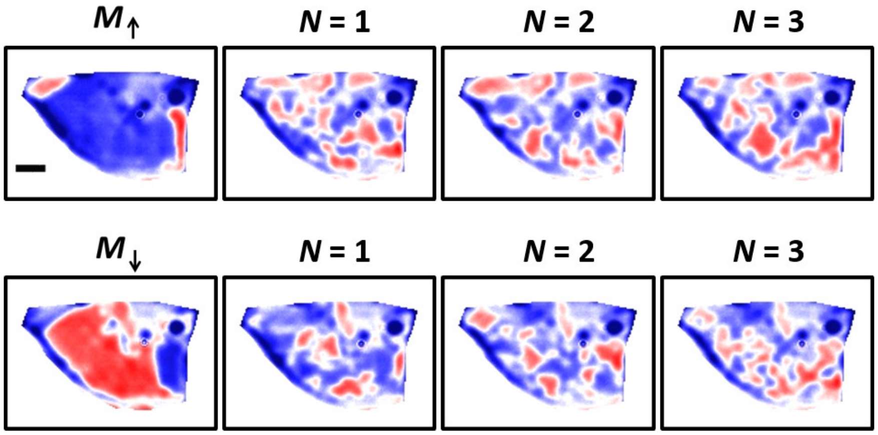


Figure S8: **Optically-induced domain formation for an isolated $CrI_3$ bulk flake (sample 1A, not shown in the main text).** Images show formation of a domain structure after consecutive single laser pulses ($N$ = 1, 2, 3) with $E = 1.67\,\text{eV}$ photon energy, linear polarization $\pi$, $\sim 30\,\text{fs}$ pulse duration and fluence $F = 7.5\,\text{mJ/cm}^2$. The measurements were performed at $T = 30\,\text{K}$. The scale bar has 5 $\mu$m length.

## 4 Domain structure and optically induced domain formation and additional AOS for heterostructure $CrI_3(10\,nm)/WSe_2(1\,L)$ (sample 2).

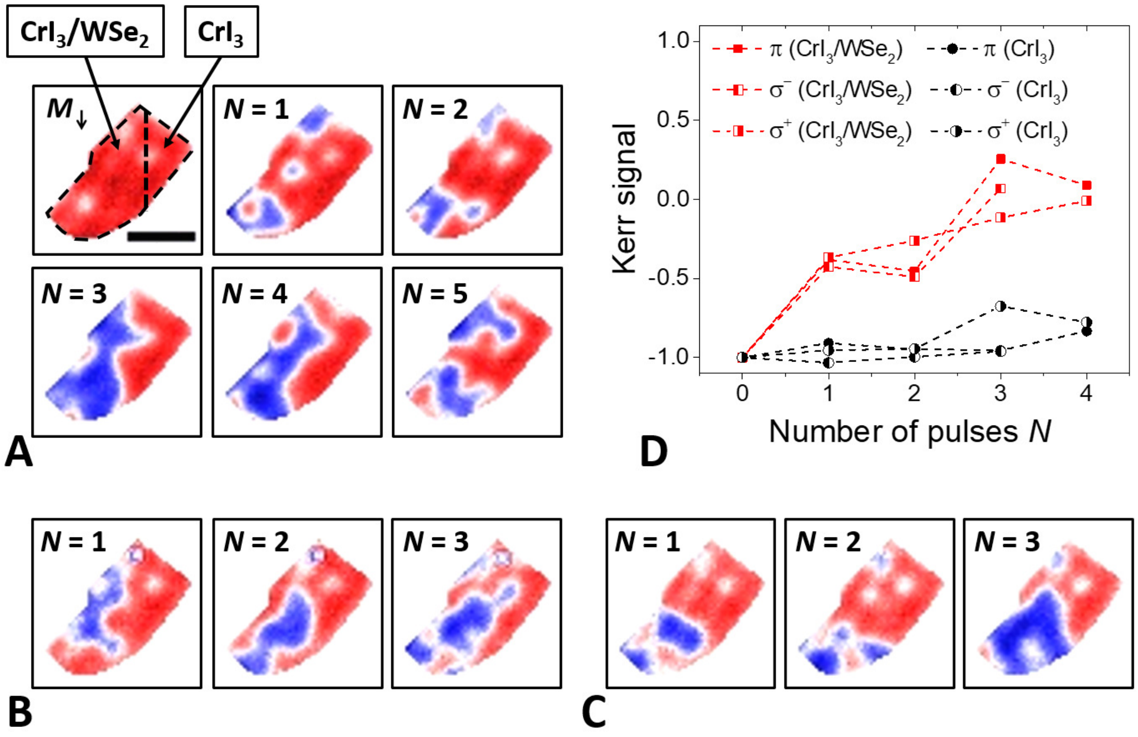


Figure S9: **Optically-induced domain formation with single pulses for a $CrI_3(10\,nm)/WSe_2(1\,L)$ heterostructure (sample 2)**. Kerr images upon excitation with **(A)** $\pi$, **(B)** $\sigma^+$ and **(C)** $\sigma^-$ polarized $N$ single pulses. For all polarizations the optical pumping starts from the $M_\downarrow$ remanent state. **(D)** The Kerr signal extracted from the domain structure images acquired after pumping with single laser pulses with different polarizations, and plotted for different parts of the flake: $CrI_3/WSe_2$ and $CrI_3$, indicated by the black dashed lines in the remanent domain state $M_\downarrow$ image. Measurements were made for $T = 35\,K$, $E = 1.67\,eV$, $\sim 30\,fs$ pulse duration and $F = 6.9\,mJ/cm^2$. The scale bar has $5\,\mu m$ length.

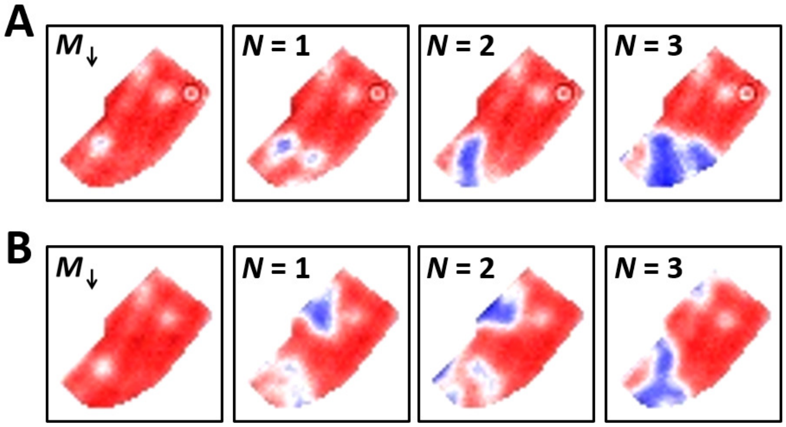


Figure S10: **Optically-induced domain formation with linearly polarized $\pi$ pulses for a $CrI_3(10\,nm)/WSe_2(1\,L)$ heterostructure (sample 2)**. Kerr images upon excitation with **(A)** $N$ single pulses and **(B)** $N$ bunches of $10^4$ pulses, starting from the $M_\downarrow$ remanent state. The pump fluence was $F = 6.5$ mJ/cm$^2$, i.e., lower than in Fig. S9. Measurements were made for $T = 35$ K, $E = 1.67$ eV, and $\sim 30$ fs pulse duration. The scale bar has 5 $\mu$m length.

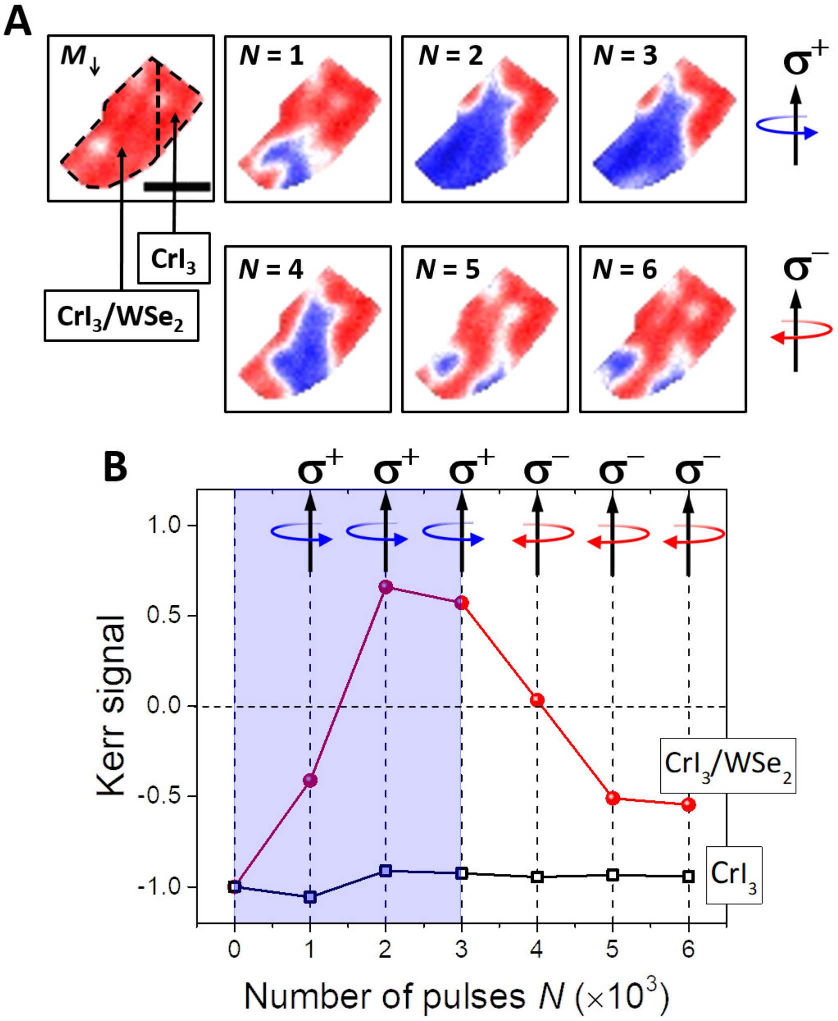


Figure S11: **AOS for a $CrI_3$(10 nm)/$WSe_2$(1 L) heterostructure (sample 2) with circularly polarized bunches of pulses**. **(A)** Kerr images upon excitation with $\sigma^+$ ($N = 1, 2, 3$ bunches of $10^3$ pulses ) and $\sigma^-$ ($N = 4, 5, 6$ bunches of $10^3$) pulses, starting from the $M_\downarrow$ remanent state. **(B)** The Kerr signal extracted from the images in **(A)** and plotted for different parts of the flake: $CrI_3/WSe_2$ (red circles) and $CrI_3$ (black squares). Measurements were made for $T = 35$ K, $E = 1.67$ eV, $\sim 30$ fs pulse duration and $F = 6.9$ mJ/cm$^2$. The scale bar has 5 $\mu$m length.

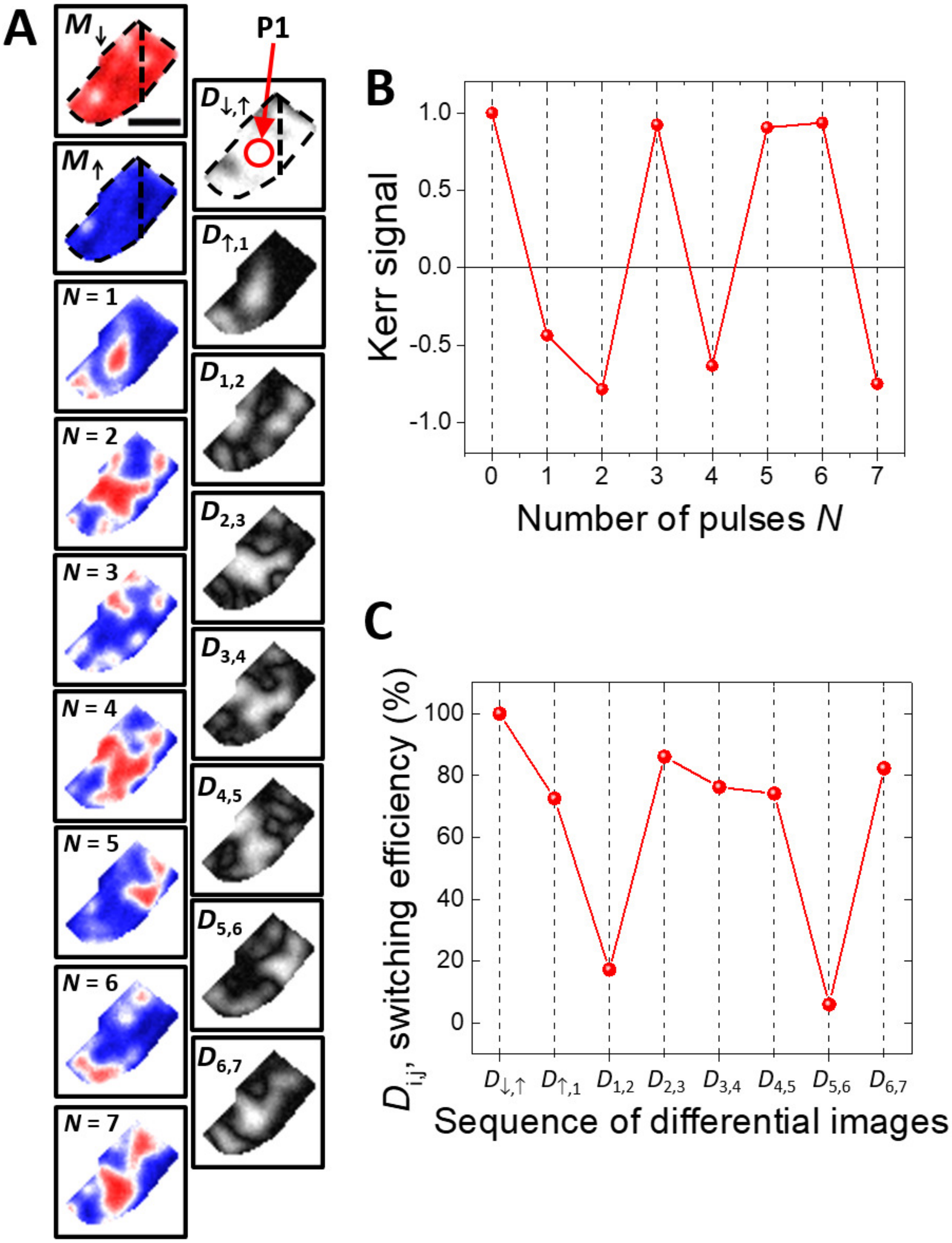


Figure S12: **AOS with single pulses for a $CrI_3$(10 nm)/$WSe_2$(1 L) heterostructure (sample 2). (A)** Kerr images and corresponding differential images $D_{i,j} = \left|(M_i - M_j)\right| / (|M_i| + \left|M_j\right|)$ upon excitation with $\pi$-polarized single pulses. **(B)** Kerr signal and **(C)** $D_{i,j}$ (switching efficiency) integrated over the area P1. Measurements were made for $T = 35$ K, $E = 1.67$ eV, $\sim 30$ fs pulse duration and $F = 7.5$ mJ/cm$^2$. The scale bar has 5 $\mu$m length.

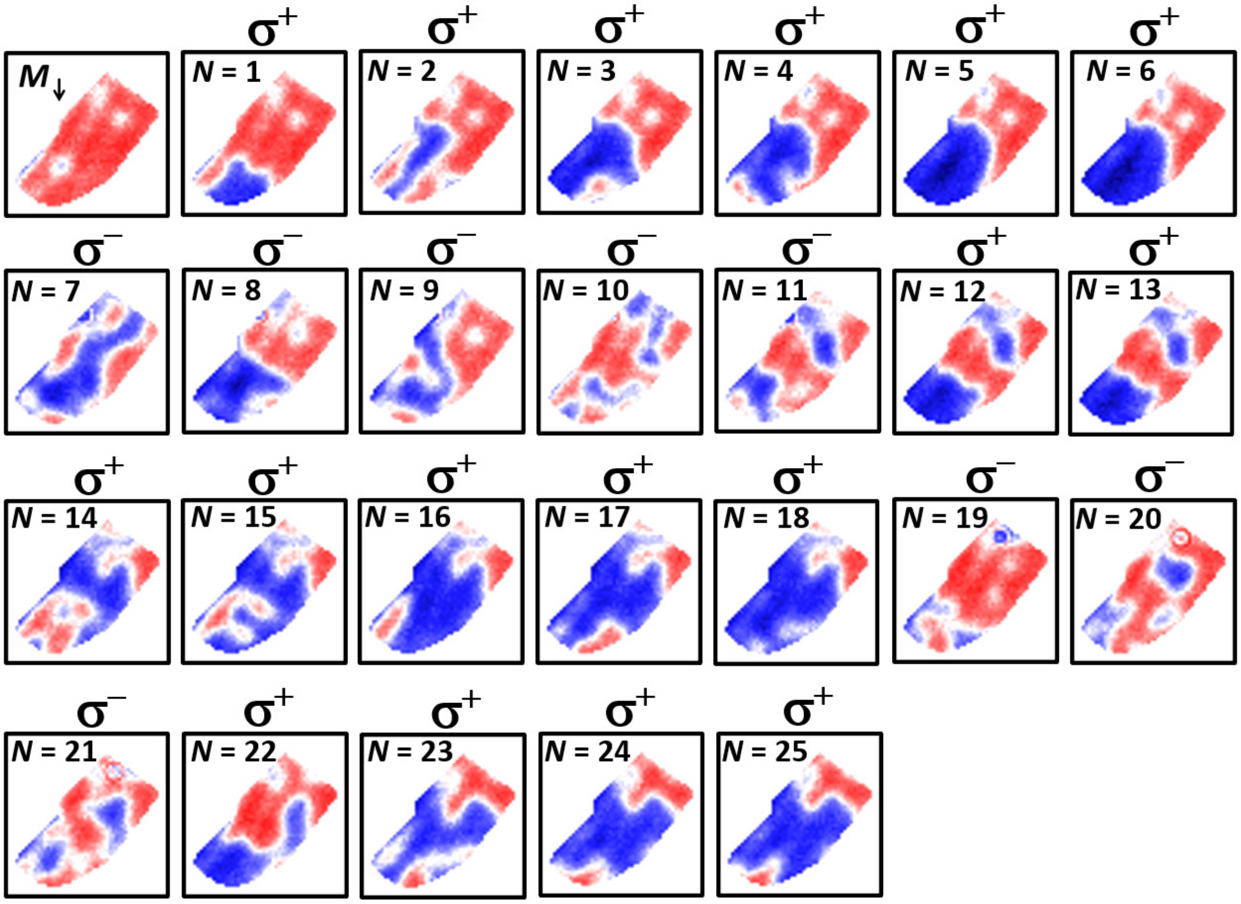


Figure S13: **HD-AOS for a $CrI_3(10\,nm)/WSe_2(1\,L)$ heterostructure (sample 2).** Kerr images upon excitation with circular polarization $\sigma^+$ and $\sigma^+$, with $N$ bunches of $10^2$ pulses, used for Fig. 2d in the main text. Measurements were made for $T = 35$ K, $E = 1.67$ eV, $\sim 30$ fs pulse duration and $F = 6.9$ mJ/cm$^2$.

## 5 Magnetization reversal and Kerr images showing the AOS for a $CrI_3(10\,nm)/WSe_2(1\,L)$ heterostructure (sample 3).

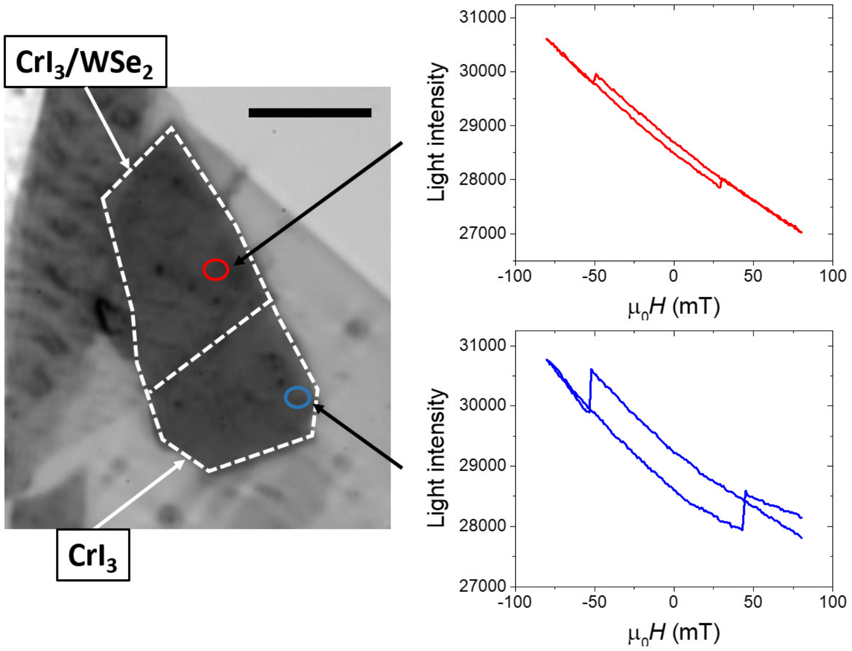


Figure S14: **Magnetization reversal of a $CrI_3(10\,nm)/WSe_2(1\,L)$ heterostructure (sample 3).** Optical image of sample 3 acquired by WFKM and hysteresis loops extracted from images at two different positions on the flake, as indicated by the red and blue circles. The $CrI_3$ flake was partially overlapped with a $WSe_2$ monolayer, as indicated by the white dashed lines. The measurements were performed at $T = 35\,K$. The scale bar has $10\,\mu m$ length.

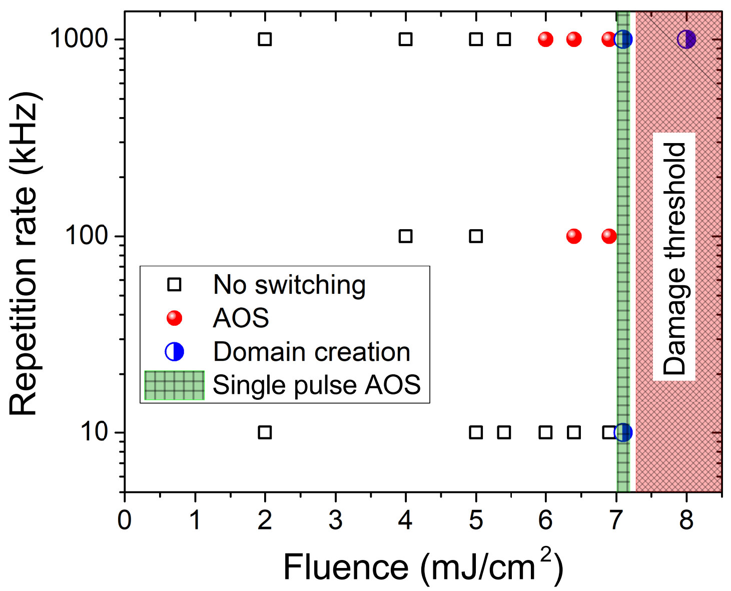


Figure S15: **Effect of the laser fluence and repetition rate on the AOS of a $CrI_3$(10 nm)/$WSe_2$(1 L) heterostructure (sample 3).** The measurements were performed at $T = 35$ K, for excitation with linearly polarized pulses with energy E = 1.67 eV and $\sim 30$ fs pulse duration. All measurements were performed for N bunches of $10^6$ pulses, except where a single pulse was used (patterned green column). Damage was observed after exposure to a single pulse with fluence of 7.3 mJ/cm$^2$.

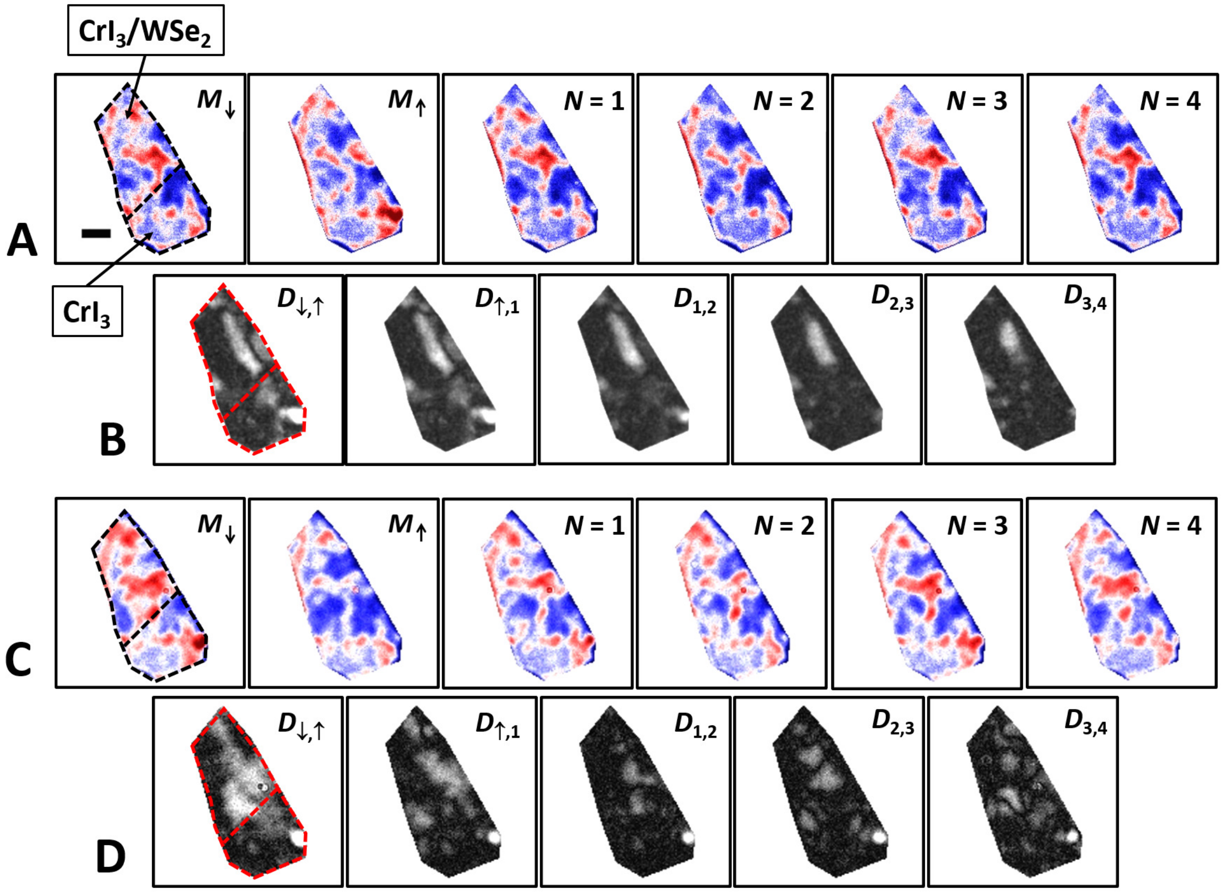


Figure S16: **AOS with linearly polarized pulses for a $CrI_3(10\,nm)/WSe_2(1\,L)$ heterostructure (sample 3). (A,C)** Kerr images and **(B,D)** differential images for the $M_\uparrow$ remanent state pumped with **(A,B)** $N$ bunches of $10^6$ pulses and **(C,D)** $N$ single pulses. Measurements were made for $T = 35\,K$, $E = 1.67\,eV$, $\sim 30\,fs$ pulse duration and fluence $F = 6.4\,mJ/cm^2$ **(A,B)** and $F = 7.1\,mJ/cm^2$ **(C,D)**. The scale bar has 5 $\mu$m length.

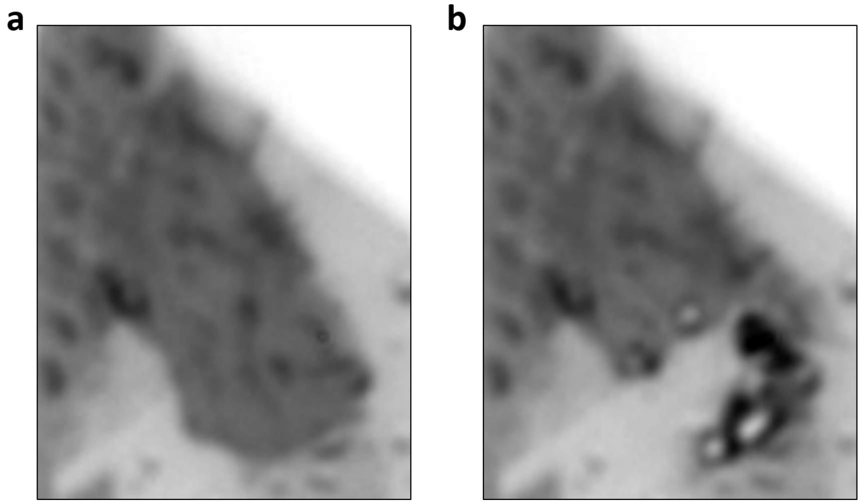


Figure S17: **Damage to a $CrI_3(10\,nm)/WSe_2(1\,L)$ heterostructure (sample 3).** Optical image before **(a)** and after **(b)** exposure to a single pulse with fluence of $7.3\,mJ/cm^2$, energy $E = 1.67\,eV$ and $\sim 30\,fs$ pulse duration.

## 6 Ultrafast atomistic spin dynamics

We model the ultrafast dynamics in $CrI_3$ with the two-temperature model [2] since $CrI_3$ behaves as a quasi-metal[3] under the influence of laser excitation. The photon energy is transferred to the free electrons, which then equilibrate with the phonon and spin baths. The spin part is treated within the atomistic spin dynamics[4–7] while the electron and phonon heat baths are described phenomenologically by the two-temperature model[2]. The coupled equations of the two-temperature model are:

$$C_{e0}T_e\frac{dT_e}{dt} = -G_{ep}(T_e - T_p) + P(t) \tag{1}$$

$$C_p\frac{dT_p}{dt} = -G_{ep}(T_p - T_e) \tag{2}$$

where $C_{e0}$ and $C_p$ are the electron and phonon heat capacities respectively, $G_{ep}$ represents the electron-phonon coupling factor, $T_p$ and $T_e$ are the phonon and electron temperatures respectively, and $P(t)$ is the time dependent laser pulse power. The electronic temperature is coupled to the magnetic system through the thermal field that enters the Landau-Lifshitz-Gilbert (LLG) equation. The laser power density takes a Gaussian form $P(t) = \frac{2F_0}{\delta t_p\sqrt{\pi/\ln 2}}\exp[(-4\ln 2)(\frac{t}{t_p})^2]$, where $F_0$ is the laser fluence (in units of energy density), $t_p$ is the temporal width of the pulse, and $\delta$ is the optical penetration depth, with an assumed value of $\delta = 10$ nm.

All simulations assume a fixed atomic spin lattice with a magnetic unit vector ($\mathbf{S}_i = \boldsymbol{\mu}_i/\mu_s$) associated with each site. The spins can precess in an effective field $\mathbf{H}_i$ according to the LLG equation

$$\frac{\partial \mathbf{S}_i}{\partial t} = -\frac{\gamma}{(1+\alpha^2)}\mathbf{S}_i \times (\mathbf{H}_i + \alpha\mathbf{S}_i \times \mathbf{H}_i) \tag{3}$$

where $\gamma$ represents the gyromagnetic ratio and $\alpha$ is the thermal bath coupling constant. The effective field $\mathbf{H}_i$ is obtained from the derivative of the Hamiltonian $\mathscr{H}$ with respect to the spin $\mathbf{S}_i$ (normalised by the magnetic moment for each site $\mu_i$ and the magnetic permeability $\mu_0$) to which is added the thermal field $\xi_i$ obtained from Langevin Dynamics. The thermal fluctuations on each site are given by a frequency independent Gaussian white noise term, the strength of the thermal fluctuations being $D = \frac{\alpha k_B T}{\gamma \mu_i}$, where $k_B T$ is the thermal energy and $\alpha$ again represents the thermal bath coupling. The total effective field may therefore be written as

$$\mathbf{H}_i = -\frac{1}{\mu_i \mu_0} \frac{\partial \mathscr{H}}{\partial \mathbf{S}_i} + \xi_i. \tag{4}$$

In our model, we assume a Hamiltonian of form

$$\mathscr{H} = -\frac{1}{2} \sum_{i,j} \mathbf{S}_i^{\alpha} \mathscr{J}_{ij}^{\alpha\beta} \mathbf{S}_j^{\beta} - \frac{1}{2} \sum_{i,j} K_{ij} (\mathbf{S}_i \cdot \mathbf{S}_j)^2 - \sum_i D_i (\mathbf{S}_i \cdot \mathbf{e})^2 \tag{5}$$

where we include higher order exchange interactions via the biquadratic exchange, which has been proved to be important for 2D magnets[4,5,8], in addition to bilinear exchange interactions and uniaxial anisotropy. Here $i$, $j$ represent the spin site index, $\alpha, \beta = x, y, z$, $\mathscr{J}_{ij}^{\alpha\beta}$ represents the exchange tensor that includes only the diagonal exchange components (the anti-symmetric exchange, e.g., the Dzyaloshinskii-Moryia interaction (DMI) is neglected in this model), $K_{ij}$ is the strength of the biquadratic exchange interaction, $D_i$ is the uniaxial anisotropy constant, and the uniaxial axis is orientated out of plane ($\mathbf{e} = (0,0,1)$). The effective field $\mathbf{H}_i$ can be calculated from the Hamiltonian to which we add thermal noise $\xi_i$ - Eq.4. This so-called Langevin thermostat produces white noise with properties determined from the Fokker-Planck equation. In the model we consider the micromagnetic dipole-dipole interaction[5] within an integration macrocell of 2nm size. The dipole fields are updated after every 100 integration time-steps. A time-step of 0.5fs is

used. We start the calculations at $T = 0$K in a saturated ferromagnetic state. No external field is applied to the system. The exchange parameterisation and magnetic anisotropy for $CrI_3$ follow that previously computed[5]. Table 1 presents the parameters used in the spin-dynamics simulations. The electron-phonon (e-ph) coupling is calculated at a specific temperature[9]. Calculations in Figure 1 in the main text assumed values of 0.1 for the damping parameter, $4.05\times10^{15}$ J m$^{-3}$ K$^{-1}$ s$^{-1}$ for the electron-phonon coupling, and a pulse width of 85 fs with a fluence of 0.04 mJ/cm$^2$.

Fig. S18 shows the ultrafast magnetisation dynamics of $CrI_3$, with domain creation and evolution following single pulse (Fig. S18A) and multi-pulse excitation (Fig. S18B) for a small heat-bath coupling. On short timescales after the excitation, the domains formed are rather small (Fig. S18C) but their size and number increase after applying multiple identical pulses (Fig. S18D). Demagnetization takes place after each pulse, as in the measurements. We next increase the laser pulse power applied to the system to 0.07 mJ/cm$^2$ (Fig. S19) to study any possible variation of the thermalization at an increased excitation energy. We observe that $CrI_3$ demagnetises almost completely after the application of the first pulse N=1 (Fig. S19A-B) and remains in a multidomain state characterised by a small value of magnetisation for longer timescales. By applying multiple pulses (N$>$ 1), the multidomain state is preserved (Fig. S19C-D). The magnetisation measured at 1 ns after the application of the laser pulse suggests that after two laser pulses there is almost no change in the magnetic state of the system. Fig. S20 summarises the magnetic domain states that are obtained for bare $CrI_3$ by changing the characteristics of the laser pulse (pulse width and pulse fluence) for two values of the heat-bath coupling constant used in the simulations: $\alpha = 0.1$ (row A) and $\alpha = 0.01$ (row B). There is no strong dependence of the domain pattern on the pulse

width which suggests that the domain formation is driven by the thermal excitation associated with the laser pulse rather than by its ultrafast character. The domain size is strongly influenced by the thermal bath coupling, with a smaller coupling leading to smaller domain size and slower dynamics. Increasing the pulse fluence, we observe that the system has compensated domains.

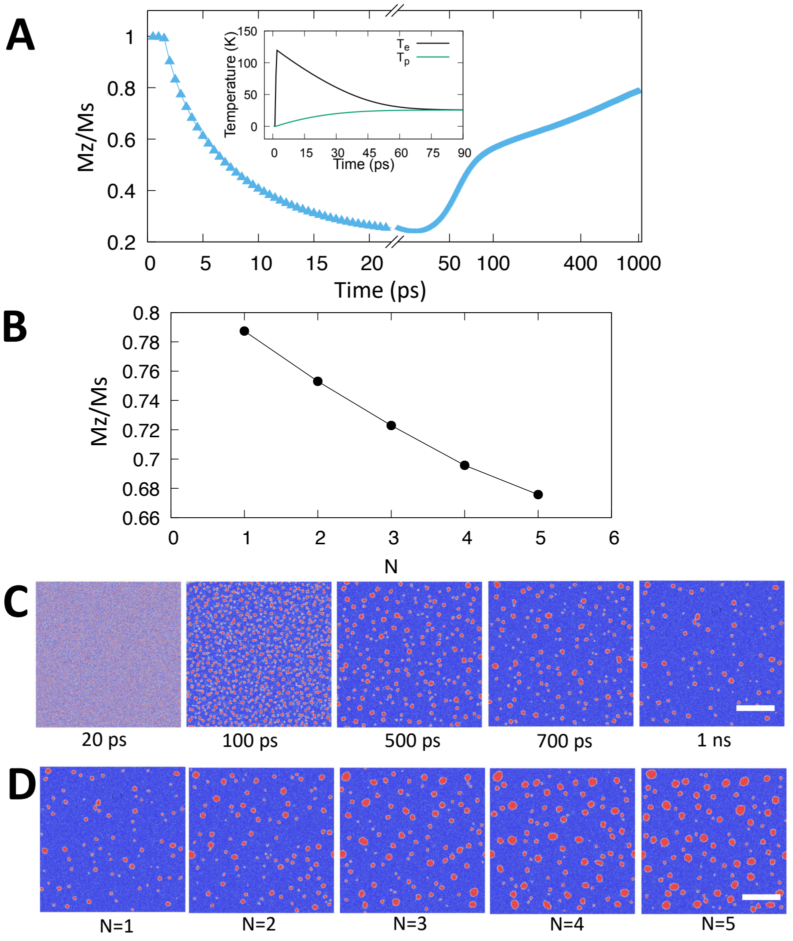


Figure S18: **Domain evolution in $CrI_3$ during laser excitation at** 0.04 **mJ/cm$^2$.** The pulse width used in this simulation is 85 fs and the fluence 0.04 mJ/cm$^2$ with a heat bath constant of 0.01 and an electron-phonon coupling factor of $1.35 \times 10^{15}$ J m$^{-3}$ K$^{-1}$ s$^{-1}$. **(A)** Time evolution of the out-of-plane magnetisation after application of the laser pulse. (Inset) Electronic and phononic temperature calculated from the two-temperature model. **(B)** Final magnetisation $M_z/M_s$ of the system extracted at 1 ns during the application of multiple laser pulses (N). **(C)** Evolution of magnetic domains following application of the laser-pulse. **(D)** Spin maps extracted every nanosecond during the application of multiple laser pulses. For each laser pulse a new random seed is chosen. To decrease the computational cost, after each pulse the system returns to 0 K in one step rather than undergoing ns timescale cooling to the heat-sink temperature. The scale bar has 100 nm length.

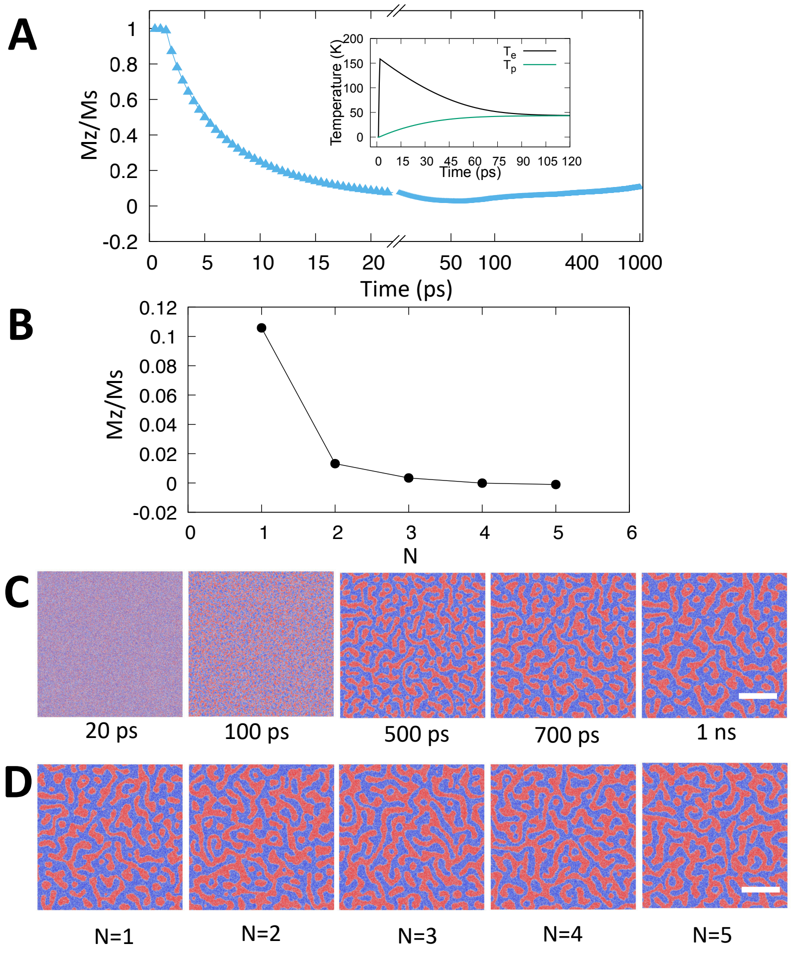


Figure S19: **Domain evolution in $CrI_3$ during laser excitation at** 0.07 **mJ/cm$^2$.** The pulse width used in this simulation is 85 fs and the fluence 0.07 mJ/cm$^2$ with a heat bath constant of 0.01 and an electron-phonon coupling factor of $1.35 \times 10^{15}$ J m$^{-3}$ K$^{-1}$ s$^{-1}$. **(A)** Time evolution of the out-of-plane magnetisation after application of the laser pulse (N=1). The inset shows the electronic and phononic temperatures calculated from the two-temperature model. **(B)** Final magnetisation of the system extracted at 1 ns during the application of multiple laser pulses. **(C)** Time-evolution of magnetic domains following application of the laser-pulse. **(D)** Spin maps extracted every nanosecond during the application of multiple laser pulses. For each laser-pulse a new random seed is chosen. To decrease the computational cost, after each pulse the system returns to 0 K in one step rather than undergoing ns timescale cooling to the heat-sink temperature. The scale bar has 100 nm length.

| Quantity | Symbol | Value | Units |
|---|---|---|---|
| Electron-heat capacity constant | $C_{e0}$ | 550 | $Jm^{-3}K^{-2}$ |
| Phonon specific heat capacity | $C_p$ | $1.5\times10^5$ | $Jm^{-3}K^{-1}$ |
| Electron-phonon coupling factor | $G_{ep}$ | $4.05\times10^{15}, 1.35\times10^{15}$ | $Jm^{-3}K^{-1}s^{-1}$ |
| Thermal bath coupling constant | $\alpha$ | $0.1, 0.01$ | |

Table 1: Magnetic parameters entering into the two temperature model, extracted from experiments in Ref.[9].

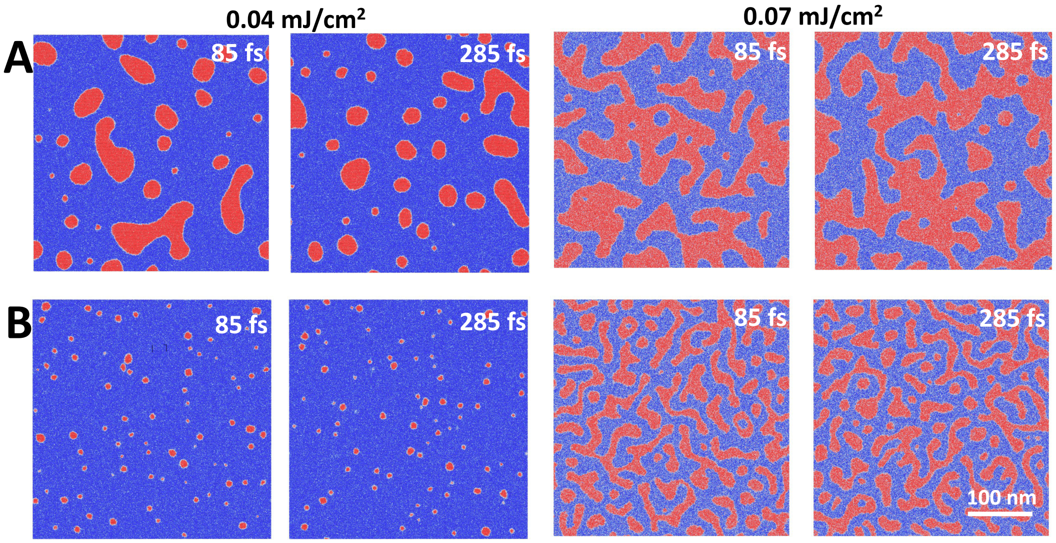


Figure S20: **Spin states simulated at 1 ns after the application of a laser pulse.** The pulse width and fluence used are 85 fs and 285 fs, and 0.04mJ/cm$^2$ and 0.07mJ/cm$^2$, respectively. **(A)** A Gilbert damping of 0.1 and electron-phonon coupling factor of $4.05\times10^{15}$ J m$^{-3}$ K$^{-1}$ s$^{-1}$ have been used, while in **(B)** the damping and electron-phonon coupling factor used are 0.01 and $4.05\times10^{15}$ J m$^{-3}$ K$^{-1}$ s$^{-1}$ respectively.

## 7 Supplementary References


1. D. Zhong, *et al.*, *Sci. Adv.* **3**, e1603113 (2017).
2. J. Chen, D. Tzou, J. Beraun, *Int. J. Heat Mass Transf.* **49**, 307 (2006).
3. M. Wu, Z. Li, T. Cao, S. G. Louie, *Nat. Commun.* **10**, 1 (2019).
4. A. Kartsev, M. Augustin, R. F. Evans, K. S. Novoselov, E. J. Santos, *npj Comput. Mater.* **6**, 1 (2020).
5. D. A. Wahab, *et al.*, *Adv. Mater.* **33**, 2004138 (2021).
6. M. Augustin, S. Jenkins, R. F. L. Evans, K. S. Novoselov, E. J. G. Santos, *Nat. Commun.* **12**, 185 (2021).
7. R. F. L. Evans, *et al.*, *J. Condens. Matter Phys.* **26**, 103202 (2014).
8. J. Macy, *et al.*, *Appl. Phys. Rev.* **8**, 041401 (2021).
9. P. Padmanabhan, *et al.*, arXiv:2010.04915 [cond-mat.mtrl-sci] (2020).
